# Maresin 1 attenuates pro‐inflammatory activation induced by β‐amyloid and stimulates its uptake

**DOI:** 10.1111/jcmm.16098

**Published:** 2020-11-22

**Authors:** Ying Wang, Axel Leppert, Shuai Tan, Bram van der Gaag, Nailin Li, Marianne Schultzberg, Erik Hjorth

**Affiliations:** ^1^ Department of Neurobiology, Care Sciences & Society Division of Neurogeriatrics Karolinska Institutet Solna Sweden; ^2^ Department of Biosciences and Nutrition Karolinska Institutet Huddinge Sweden; ^3^ Department of Medicine Clinical Pharmacology Group Karolinska University Hospital Solna Sweden

**Keywords:** Alzheimer's disease, chemokines, cytokines, flow cytometry, human, monocyte‐derived microglia, NF‐κB, pro‐resolving, resolution of inflammation, THP‐1

## Abstract

Alzheimer's disease (AD) is the most common dementia, characterized by pathological accumulation of β‐amyloid (Aβ) and hyperphosphorylation of tau protein, together with a damaging chronic inflammation. The lack of effective treatments urgently warrants new therapeutic strategies. Resolution of inflammation, associated with beneficial and regenerative activities, is mediated by specialized pro‐resolving lipid mediators (SPMs) including maresin 1 (MaR1). Decreased levels of MaR1 have been observed in AD brains. However, the pro‐resolving role of MaR1 in AD has not been fully investigated. In the present study, human monocyte‐derived microglia (MdM) and a differentiated human monocyte cell line (THP‐1 cells) exposed to Aβ were used as models of AD neuroinflammation. We have studied the potential of MaR1 to inhibit pro‐inflammatory activation of Aβ and assessed its ability to stimulate phagocytosis of Aβ_42_. MaR1 inhibited the Aβ_42_‐induced increase in cytokine secretion and stimulated the uptake of Aβ_42_ in both MdM and differentiated THP‐1 cells. MaR1 was also found to decrease chemokine secretion and reduce the associated increase in the activation marker CD40. Activation of kinases involved in transduction of inflammation was not affected by MaR1, but the activity of nuclear factor (NF)‐κB was decreased. Our data show that MaR1 exerts effects that indicate a pro‐resolving role in the context of AD and thus presents itself as a potential therapeutic target for AD.

## 1 INTRODUCTION

Alzheimer's disease (AD) is the leading cause of dementia characterized by neuronal loss, and pathological accumulation of neurotoxic β‐amyloid (Aβ) and hyperphosphorylated tau proteins, together with damaging chronic inflammation as indicated by activated microglia, which are the resident immune cells in the central nervous system (CNS) where they are important players in health as well as disease. In health, microglia execute supporting functions for the nervous tissue by trophic support, synapse maintenance and phagocytic removal of molecular and cellular debris, as well as surveillance of the tissue for pathogenic threats.^1^, ^2^, ^3^ In AD, microglia are activated by an overabundance of Aβ, a reaction that is further augmented due to the increase in misfolded and aggregated forms of this peptide, and thus initiate an innate immune response that contributes to the pathogenesis by increasing neurotoxic pro‐inflammatory mediators while neuroprotective anti‐inflammatory mediators are decreased, and oxidative stress increased.^4^, ^5^, ^6^, ^7^ Furthermore, phagocytosis is impaired,^8^, ^9^, ^10^ thus limiting the ability of microglia to decrease the amyloid burden.

Considering the harmful consequences of chronic inflammation,^5^, ^11^, ^12^, ^13^, ^14^ it makes sense that the inflammatory response should be ended as soon as the pathogenic threat is neutralized. Under normal physiological conditions, an inflammation is cleared by resolution,^15^ which is an active process wherein the beneficial aspects of inflammation are increased while the damaging ones are decreased. This process is associated with the restoration and regeneration that occurs in healing. Thus, the damaged area where inflammation has acted is returned to homeostasis. Resolution of inflammation is mediated by specialized pro‐resolving lipid mediators (SPMs) including lipoxins, resolvins, maresins and protectins are derived from n‐3 and n‐6 fatty acids.^15^, ^16^, ^17^, ^18^ SPMs down‐regulate the inflammatory response, normalize chemokine gradients, facilitate the apoptosis of polymorphonuclear leucocytes and initiate the regeneration of local tissue by trophic activity and phagocytosis of molecular and cellular debris.^15^, ^19^, ^20^, ^21^ Most research on resolution has been focused on the periphery, while the CNS has received less attention. There is, however, evidence for a failed and dysfunctional resolution in the AD brain, which can contribute to pathology and pathogenesis, indicated by decreased levels of SPMs in the hippocampus, entorhinal cortex and cerebrospinal fluid (CSF),^20^, ^22^, ^23^ together with alterations in the levels of receptors for SPMs.^24^, ^25^ An impaired resolution in AD means that the brain is not only continuously exposed to the debilitating effects of chronic neuroinflammation but is also deprived of important trophic support and maintenance. One of the SPMs shown to be decreased in AD brains is maresin 1 (MaR1).^20^ MaR1, derived from omega‐3 fatty acid docosahexaenoic acid (DHA), was first detected by Serhan *et al* in mouse peritonitis exudates, using liquid chromatography–tandem mass spectrometry,^15^ and subsequently, the pro‐resolving roles of MaR1 have been identified in several disease models.^26^, ^27^, ^28^, ^29^, ^30^, ^31^

AD is a disabling disease afflicting an estimated 40 million people worldwide.^32^ Societal costs amounted to about € 72 500 per person per year for residential care in 2011.^33^ However, there is currently no treatment that can reverse the progression of the disease (see ^34^). New therapeutic strategies are therefore urgently needed. Promoting the progression to resolution, leading to reduced inflammation and at the same time stimulating regeneration may be a successful therapeutic strategy for AD.

Considering the decrease in MaR1 levels in AD, and the promising results from previous in vitro and in vivo studies,^20^, ^23^, ^35^ MaR1 is a candidate substance for re‐establishing the failed resolution in AD. However, the molecular mechanisms of action of MaR1 in the brain and in AD are not yet fully known and further studies are necessary before clinical trials in humans can be considered. The aim of the present study is to investigate the effects of MaR1 in a model of Aβ_42_‐induced inflammation in two human in vitro models, with the hypothesis that MaR1 will stimulate a phenotype switch, resolve the inflammation, increase phagocytosis of Aβ_42_ and improve cell survival and that these effects will be associated with reduced activity of intracellular pro‐inflammatory signalling pathways.

## 2 MATERIALS AND METHODS

### 2.1 Materials

Human THP‐1 and primary human monocytes were obtained from LGC Standards (Teddington, UK) and Lonza (Basel, Switzerland), respectively. RPMI‐1640 cell culture medium, foetal bovine serum, N2 supplement, stable trypsin replacement enzyme, Pen‐Strep, BCA assay kit, QuantaRed^TM^ enhanced chemifluorescent horseradish peroxidase substrate, phosphatase inhibitor cocktail and Alexa Fluor 546 secondary antibodies were purchased from Thermo Fisher Scientific (Stockholm, Sweden). Cell culture plates were obtained from VWR (Stockholm, Sweden). Phorbol 12‐myristate 13‐acetate (PMA), isopropyl‐β‐D‐thiogalactoside, β‐mercaptoethanol, okadaic acid, 1% protease inhibitor cocktail, 3‐isobutyl‐1‐methylxanthine and radioimmunoprecipitation assay (RIPA) buffer were purchased from Sigma Chemical Co. (Stockholm, Sweden). CD14 microbeads, whole blood column kit, interleukin (IL)‐34, granulocyte‐macrophage colony‐stimulating factor (GM‐CSF) and M‐CSF were obtained from Miltenyi Biotec (Lund, Sweden). Recombinant human nerve growth factor (NGF)‐β protein was purchased from Alomone Labs (Jerusalem, Israel). MaR1 was purchased from Cayman Chemical (Stockholm, Sweden). Prior to use, RPMI‐1640 medium was added to reconstitute MaR1 after ethanol evaporation under a N_2_ stream. Antibodies raised against phospho‐p38 mitogen‐activated protein kinase (MAPK) (Thr180/Tyr182), p38 MAPK, phospho‐p44/42 MAPK (Thr202/Tyr204), p44/42 MAPK, phospho‐protein kinase B (Akt) (Ser473), Akt and SAPK/c‐Jun N‐terminal kinase (JNK), were obtained from Cell Signaling Technology (Stockholm, Sweden), antibodies against phospho‐SAPK/JNK (pT138/pY185) were purchased from BD Biosciences (Stockholm, Sweden), and antibodies against ionized calcium‐binding adapter molecule 1 (Iba‐1) were obtained from Wako Chemicals Europe (Täby, Sweden). Fluor 647‐labelled antibodies against human CD40 and CD163, phycoerythrin‐labelled antibodies against human CD86 and CD200R, and the corresponding isotype controls, were purchased from Biolegend (London, United Kingdom). Blocking buffer, total protein stain kit, donkey anti‐mouse IgG and donkey anti‐rabbit IgG were obtained from LI‐COR (Lincoln, NE, USA). Human tumour necrosis factor (TNF)‐α, IL‐1β, IL‐6, soluble IL‐6 receptor alpha (IL‐6Rα) and IL‐1 receptor type II enzyme‐linked immunosorbent assay (ELISA) kits and recombinant human monocyte chemoattractant protein (MCP)‐1 protein were purchased from R&D Systems (Abingdon, United Kingdom). General lipopolysaccharide (LPS) ELISA kit was obtained from Biorbyt (Cambridge, United Kingdom). V‐PLEX human chemokine panel kit detecting eotaxin‐1, eotaxin‐3, IL‐8, interferon γ‐induced protein 10 (IP‐10), MCP‐1, MCP‐4, macrophage‐derived chemokine (MDC), macrophage inflammatory protein (MIP)‐1α, MIP‐1β and thymus and activation regulated chemokine (TARC), and V‐PLEX human pro‐inflammatory panel 1 kit detecting TNF‐α, IL‐1β and IL‐6 were obtained from Meso Scale Discovery (Rockville, US). THP1‐Lucia^TM^ nuclear factor (NF)‐κB cells, Normocin (a formulation of three antibiotics active against mucoplasma, bacteria and fungi), Zeocin and QUANTI‐Luc were purchased from InvivoGen (Toulouse, France). Cytotoxicity detection kit was obtained from Roche (Solna, Sweden), and human Fluor 488‐labelled Aβ_42_ from AnaSpec (Stockholm, Sweden). DEAE cellulose and Superdex30 PG column (26/600) were obtained from GE Healthcare (Danderyd, Sweden).

### 2.2 Cell culture and stimulations

Human primary monocytes from adult healthy donors were purchased or isolated from fresh blood samples obtained from adult healthy volunteers who had given informed consent to participate in the study. All blood collection and experimental procedures were performed in compliance with the protocols approved by the Regional ethical review board in Stockholm (2019‐04340). In brief, monocytes were positively selected from whole blood samples using CD14 + microbeads and were isolated using a whole blood column kit, and subsequently plated at a density of 10^5^ cells/cm^2^ in 24‐well plates. To induce differentiation the monocytes were incubated at standard humidified culture condition (37°C in 5% CO_2_) for 10 days in RPMI‐1640 Glutamax with 1% penicillin/streptomycin, 1% N2 supplement and a mixture of the following human recombinant proteins: M‐CSF (10 ng/mL), GM‐CSF (10 ng/mL), NGF‐β (10 ng/mL), MCP‐1 (100 ng/mL) and IL‐34 (100 ng/mL).

THP‐1 cells and THP1‐Lucia^TM^ NF‐κB cells were maintained in RPMI‐1640 culture medium supplemented with 10% heat‐inactivated foetal bovine serum of EU origin at +37°C in 5% CO_2_, and 50 nM β‐mercaptoethanol was applied to THP‐1 cells, while 100 μg/mL Normocin, 100 U/mL Pen‐Strep and 100 μg/mL Zeocin were added to the THP1‐Lucia^TM^ NF‐κB cells. Cells were sub‐cultured 2‐3 times per week. The THP‐1 and THP1‐Lucia^TM^ NF‐κB cells were differentiated into macrophages (hereafter called d‐THP‐1 and d‐THP1‐Lucia^TM^ NF‐κB cells, respectively) by treatment with 50 and 5 ng/mL PMA in serum‐free medium for 72 hours, respectively. The THP‐1 cells were seeded in 6‐well plates at a density of 6 × 10^4^ cells/cm^2^, while THP1‐Lucia^TM^ NF‐κB cells were seeded in 96‐well plates at 1.5 × 10^5^ cells/cm^2^. A wash‐out of the differentiation medium with PMA‐free medium was performed at least 3 hours prior to experiments.

To analyse the effects of MaR1 on Aβ_42_‐induced pro‐inflammatory reactions, the MdM and d‐THP‐1 were incubated for 2 or 24 hours with 5 μM Aβ_42_ with or without 5 μM MaR1. At the end of the experiments, the supernatants were collected for assessment of cytokines released from MdM and d‐THP‐1. The d‐THP‐1 cells were also analysed with regard to cell viability and supernatant levels of cytokine receptors and chemokines. d‐THP‐1 cells were harvested for analysis of surface biomarkers and kinase activation.

To assess the effects of MaR1 on phagocytosis of Aβ_42_, MdM were incubated for 90 minutes with 1 μg/mL HiLyteFluor 488‐conjugated Aβ_42_ in the presence or absence of 5 μM MaR1, while d‐THP‐1 cells were incubated for 20 minutes with 1 or 5 μg/mL HiLyteFluor 488‐conjugated Aβ_42_ with or without 5 μM MaR1. Prior to flow cytometry, the d‐THP‐1 cells were photographed in a fluorescent microscope (Zeiss Axiovert 200M) to visualize the uptake of Aβ_42_.

To evaluate the effect of MaR1 on Aβ_42_‐induced NF‐κB activation, d‐THP1‐Lucia^TM^ NF‐κB cells were incubated for 24 hours with vehicle or 5 μM Aβ_42_ with or without addition of 5 μM MaR1, and the culture medium was collected for analysis of NF‐κB activation.

### 2.3 Preparation of Aβ_42_ monomers and detection of LPS contamination

Monomers of methionine (Met)‐Aβ_42_ were used to induce AD‐like inflammation in d‐THP‐1 cells. As the technique for production and purification was improved to produce wild‐type (WT) Aβ_42_, the MdM were all treated with WT Aβ_42_ monomers. Previous publications have shown that the biological effects of Met‐ and WT Aβ_42_ monomers are not significantly different.^36^ The production and purification of Met‐Aβ_42_ monomers (hereafter referred to as Aβ_42_) was performed as previously described.^36^ Briefly, recombinant Aβ_42_ was expressed in BL21*(DE3) pLysS *E coli* cells. At an OD_600_ of ~0.9, 0.5 mM isopropyl‐β‐D‐thiogalactoside (IPTG) was added for incubation overnight at +20°C, followed by centrifugation at 7300×g and storage of the cell pellets at −20°C until use. After thawing, the cells were lysed by sonication and inclusion bodies were cleared from soluble proteins by an additional round of sonication. Aβ_42_ was solubilized in 8 M urea and further purified using DEAE cellulose. The eluate was lyophilized, dissolved in 6 M Gdn‐HCl and the separation of monomeric Aβ_42_ peptide fractions from larger aggregates was performed using a Superdex30 PG column (26/600) and phosphate‐buffered saline (PBS), pH 7.4 buffer (GE Healthcare Life Sciences, UK). The concentration of Aβ_42_ monomers was calculated using an extinction coefficient of 1.424 M/cm for Abs_280‐300 nm_.

The production and purification of WT Aβ_42_ monomers was performed as previously described.^37^ Briefly, the fusion protein tag N‐terminal (NT)*_FlSp_‐Aβ_42_ was expressed in BL21*(DE3) pLysS *E coli* cells and purified using immobilized metal ion affinity chromatography (IMAC) (GE Healthcare Life Sciences, UK). The cleavage of NT*_FlSp_‐Aβ_42_ was conducted using tobacco etch virus (TEV) protease. Finally, the solution containing a mixture of TEV, NT*_FlSp_ and Aβ_42_ was applied to a Superdex30 PG column (26/600) to isolate Aβ_42_ monomers. As Aβ_42_ monomers were produced by *E coli* cells, LPS contamination was evaluated by an LPS ELISA kit with a detection range of 3.12 to 200 ng/mL. Aβ_42_ stock solution from six purifications was diluted to a working concentration of 5 µM by diluent buffer (supplied by the kit). The LPS concentrations were determined according to the distributor's instruction. LPS was not detectable in neither of the six batches (four of Met‐Aβ_42_ monomers and two of WT Aβ_42_ monomers) (see Table S1). Purified Aβ_42_ monomers were aliquoted in low‐binding tubes and stored at −20℃. Before use, Aβ_42_ was slowly thawed on ice to avoid aggregation.

### 2.4 Immunocytochemistry

Immunocytochemistry for the macrophage and microglial marker Iba‐1 was performed on d‐THP‐1 cells. In brief, the cells were fixed with 4% para‐formaldehyde (PF) in PBS. After blocking by serum, the cells were incubated with Iba‐1 antibodies (dilution 1:100) at +4°C overnight, rinsed in PBS for 15 minutes and then incubated at room temperature for 1 hour with Alexa Fluor 546 secondary antibodies (dilution 1:1000). The cell nuclei were stained with 4',6‐diamidino‐2‐phenylindole (DAPI). Microscopy was performed using a Zeiss Fluorescence microscope, and images captured with a Nikon Ds‐Fi1 camera controlled by NIS‐Elements D software (both from Bergman‐Labora, Sweden).

### 2.5 Viability assessment

Cell death was analysed by LDH assay using a cytotoxicity detection kit according to the manufacturer's instructions. The absorbance was measured at 340 nm. The absorbance of the cell culture supernatant was obtained by subtracting the absorbance of the negative control. The appearance of the d‐THP‐1 cells was evaluated in 10× magnification using a light microscope (EVOS Cell Imaging System, Thermo Fisher Scientific, Stockholm, Sweden).

### 2.6 Cytokine and IL‐6Rα assays

Concentrations of TNF‐α, IL‐1β, IL‐6 and IL‐6Rα in d‐THP‐1 culture supernatants were determined using ELISA kits according to the instructions supplied with the kits, with the exception that QuantaRed^TM^ fluorescent detection agent was used. The intensity of the emitted fluorescence at 575 nm (10 nm bandwidth) after excitation at 620 nm (10 nm bandwidth), with a gain of 60 V, was measured using a plate reader (Tecan Safire^2^, Tecan Nordic, Stockholm, Sweden). The cytokine concentrations were extrapolated from the standard curve within the recommended range for TNF‐α (15.6‐1000 pg/mL), IL‐1β (3.9‐250 pg/mL), IL‐6 (9.4‐600 pg/mL) and IL‐6Rα (31.2‐2000 pg/mL).

The levels of TNF‐α, IL‐1β and IL‐6 in the MdM culture supernatants were assessed using a V‐PLEX Meso Scale human pro‐inflammatory panel. The assay was performed according to the instructions supplied with the panel. In brief, samples and standards were incubated for 2 hours in a 96‐well plate with capture antibodies. After three washes in wash buffer, a detection antibody mixture was added for another 2 hours of incubation. The plate was then washed and after addition of reading buffer analysed in a Meso Scale Quickplex SQ120 (Maryland, US). The concentrations were extrapolated from the standard curve within the recommended range for TNF‐α (0.04‐248 pg/mL), IL‐1β (0.04‐375 pg/mL), and IL‐6 (0.06‐488 pg/mL).

### 2.7 Chemokine detection

The levels of the chemokines eotaxin‐1, eotaxin‐3, IL‐8, IP‐10, MCP‐1, MCP‐4, MDC, MIP‐1α, MIP‐1β and TARC, were determined in culture supernatants using a V‐PLEX Meso Scale human chemokine panel according to the instructions supplied with the panel (see section 2.6). The detection ranges were as followed: eotaxin‐1 (0.44‐1790 pg/mL), eotaxin‐3 (1.39‐5710 pg/mL), IL‐8 (16.3‐66700 pg/mL), IP‐10 (0.58‐2370 pg/mL), MCP‐1 (0.13‐544 pg/mL), MCP‐4 (0.17‐683 pg/mL), MDC (2.28‐9350 pg/mL), MIP‐1α (0.27‐1090 pg/mL), MIP‐1β (0.31‐1280 pg/mL) and TARC (0.46‐1870 pg/mL).

### 2.8 Western blot analysis

Analysis of phosphorylation of p38, p44/42, Akt and JNK was performed by Western blotting. Cells were washed with ice‐cold PBS, pH 7.4, and then lysed with RIPA buffer supplemented with 1% protease inhibitor and 1% phosphatase inhibitor cocktails, 0.5 nM/L 3‐isobutyl‐1‐methylxanthine and 2 nM okadaic acid. Proteins were separated by sodium dodecyl sulphate–polyacrylamide gel (SDS‐PAGE) electrophoresis in a PROTEAN II XL^TM^ system (Bio‐Rad, Stockholm, Sweden), in a 10% gel. The voltage for electrophoresis was 120 V for 30 minutes and then 160 V for 5 hours. The proteins were transferred to 0.2 µm nitrocellulose membranes (Bio‐Rad, Stockholm, Sweden) under 85 mA current overnight in a Transblot^TM^ cell (Bio‐Rad). The membranes were incubated with blocking buffer and then with primary antibodies, followed by rinsing and incubation with anti‐mouse and anti‐rabbit secondary antibodies labelled with near‐infrared fluorescent molecules IRDye800CW and IRDye680RD. Activation of p38 MAPK, p44/42 MAPK, Akt and JNK was assessed using a 1:200 dilution of antibodies specific for the phosphorylated (p‐) forms of these proteins, and a 1:800 dilution of antibodies recognizing both phosphorylated and unphosphorylated forms, that is total‐ (t‐). The dilution for secondary antibodies was 1:15 000. The blots were scanned in the Odyssey Infrared Imaging System (Li‐COR Biosciences, Sweden) and analysed using the accompanying Image Studio software. The data on protein phosphorylation were obtained by calculating the ratio between the median signal intensity for each phosphorylated form and the corresponding intensity of the total form.

### 2.9 Phagocytosis

For assessment of phagocytosis, MdM or d‐THP‐1 cells were incubated with HilyteFluor 488‐conjugated Aβ_42_ with or without addition of MaR1 as described in section 2.2. The cells were rinsed twice in PBS to stop the treatment, imaged in the microscope and harvested using a stable trypsin replacement enzyme (TryPLE, ThermoFisher, Sweden). The cells were fixed with 4% PF in PBS at room temperature for 45 minutes, and then washed twice by adding 1.5 mL PBS and centrifuged at 1500*g* for 10 minutes and then resuspended in 200 μL PBS. Phagocytosis was analysed by flow cytometry with a BD Accuri C6 Plus instrument (BD Biosciences, USA). The proportion of HiLyteFluor 488‐conjugated Aβ_42_‐positive cells was obtained by determining the percentage of cells showing a signal in the FITC‐channel exceeding the signal of the vehicle control. Flow cytometric data were analysed by the supplied BD Accuri C6 Plus software (v1.0).

### 2.10 NF‐κB activation

d‐THP1‐Lucia^TM^ NF‐κB cells were incubated for 24 hour with vehicle, 5 μM Aβ_42_ or 5 μM Aβ_42_ + 5 μM MaR1. Subsequently, 20 μL of the cell culture supernatant from each treatment were transferred to a 96‐well plate and combined with 50 μL of QUANTI‐Luc assay solution. The luminescence was measured immediately in a TECAN Safire^2^ plate reader (TECAN, Sweden).

### 2.11 Surface biomarkers

The levels of the membrane‐associated cellular biomarkers CD40, CD86, CD163 and CD200R were assessed by flow cytometry. The cells were incubated for 24 hours with vehicle, 5 μM Aβ_42_ or 5 μM Aβ_42_ + 5 μM MaR1, harvested and fixed as described in Section 2.9. Cell suspensions were incubated with fluorophore‐conjugated antibodies overnight at + 4℃. The working dilutions of antibodies and corresponding isotypes are listed in Table S2. The cells were washed once and resuspended in 200 μL PBS and analysed with a BD Accuri C6 Plus flow cytometer. The percentage of labelled cells for each marker was determined as the proportion of cells with a signal that was stronger than that for the isotype control. Flow cytometric data were analysed by the BD Accuri C6 Plus software (v1.0).

### 2.12 Statistical analysis

Statistical analyses were conducted using Statistica 12 (Dell Software, Aliso Viejo, USA). All data were normalized to the mean of each individual experiment. Kruskal–Wallis ANOVA was used to test for group differences, with the built‐in *post hoc* test, or manually with Mann‐Whitney with Bonferroni correction for multiple comparisons, used to test for differences between treatments. A *P* value of <0.05 was considered statistically significant.

## 3 RESULTS

In the present study, we have analysed the effects of MaR1 on Aβ_42_‐induced pro‐inflammatory cytokine secretion and uptake of Aβ_42_ in two models of human microglia, MdM and d‐THP‐1 cells. The effects of MaR1 on Aβ_42_‐induced cell death, chemokine expression, phenotype changes and intercellular pathway activation were investigated in d‐THP‐1 cells. The experiments were repeated 5 to 14 times.

### 3.1 Morphological changes of THP‐1 monocytes during differentiation and after Aβ_42_ treatment

**Figure 1:**
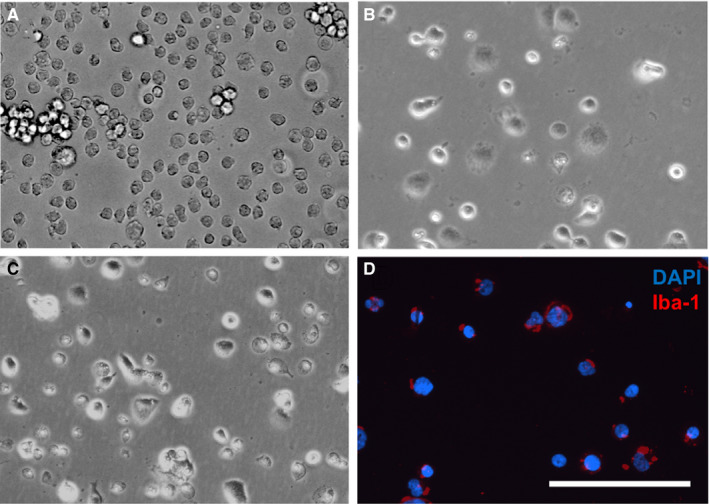
(A‐D) Morphological changes of THP‐1 monocytes during differentiation and after Aβ_42_ treatment. (A) Undifferentiated human THP‐1 monocytes are free‐floating and have a rounded shape. (B) Incubation of the cells for 72 h with 50 ng/mL PMA resulted in differentiation to a macrophage phenotype (d‐THP‐1 cells). The cells have attached to the culture plates and become elongated and flattened. (C) After incubation of the d‐THP‐1 cells with 5 μmol/L Aβ_42_ for 24 h, the cells clustered together and displayed a heterogeneous morphology with different sizes and shapes. (D) The micrograph shows staining for Iba‐1 (red fluorescence) in untreated d‐THP‐1 cells. The cell nuclei were stained by DAPI (blue fluorescence). All micrographs are of the same magnification and the bar indicates 200 µm. Aβ = β‐amyloid; DAPI = 4',6‐diamidino‐2‐phenylindole; Iba‐1 = ionized calcium‐binding adapter molecule 1; PMA = phorbol 12‐myristate 13‐acetate

Undifferentiated THP‐1 monocytes exhibited a round single‐cell morphology without attachment to the cell culture flask (Figure 1A), and after differentiation by exposure to PMA, the cells started to adhere to the culture plate and converted to mature macrophages (d‐THP‐1 cells) with an elongated and flattened morphology (Figure 1B). Incubation of the d‐THP‐1 cells with Aβ_42_ altered the morphology to an irregular shape (Figure 1C). Immunocytochemistry for the microglia/macrophage marker Iba‐1 showed staining of the d‐THP‐1 cells (Figure 1D). Iba‐1 is mainly expressed in mature macrophages and microglia and thereby the staining confirms differentiation of the THP‐1 cells into macrophages.

### 3.2 MaR1 decreased Aβ_42_‐induced secretion of pro‐inflammatory cytokines

The effect of MaR1 on the release of Aβ_42_‐induced pro‐inflammatory cytokines was investigated. d‐THP‐1 cells and MdM were incubated for 24 hours with 5 μM Aβ_42_ alone or together with 5 μM MaR1. At the end of the experiments (in total 5 for MdM and 14 for d‐THP‐1), the supernatants were collected for assessment of TNF‐α, IL‐1β and IL‐6 using ELISA.

**Figure 2:**
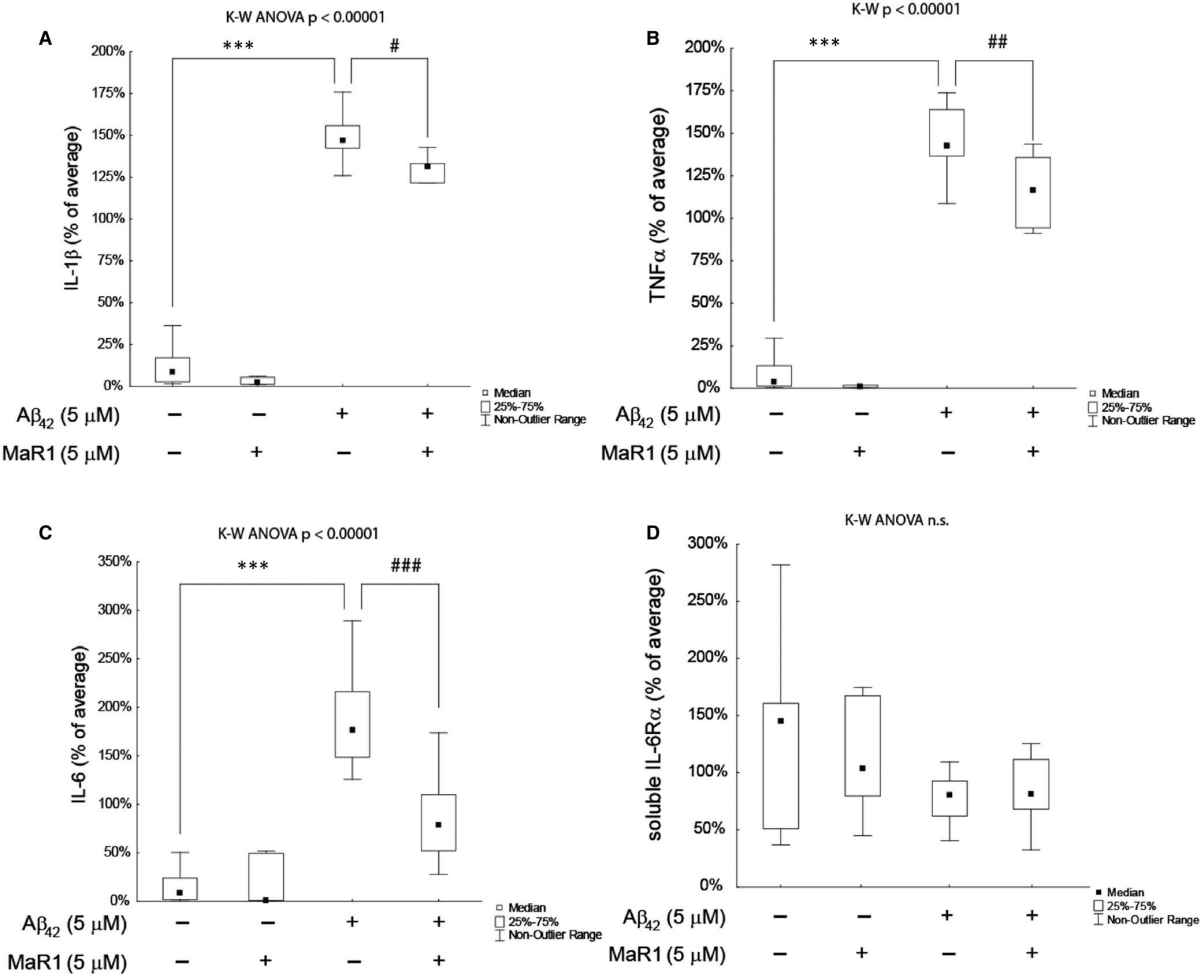
(A‐D) MaR1 reduced Aβ_42_‐induced secretion of pro‐inflammatory cytokines in d‐THP‐1 cells. Differentiated THP‐1 (d‐THP‐1) cells were incubated with vehicle, 5 µM Aβ_42_, 5 µmol/L MaR1 or 5 µM Aβ_42_ + 5 µM MaR1 for 24 h and the supernatants were analysed by ELISA. A total of 14 experiments were performed. MaR1 reduced the Aβ_42_‐induced increase in interleukin (IL)‐1β (A), tumour necrosis factor (TNF)‐α (B) and IL‐6 (C). The levels of IL‐6 receptor (R) α (D) were not affected by Aβ_42_ nor MaR1. Analysis of variance (ANOVA) was performed with the non‐parametric Kruskal–Wallis (K‐W) test, using the built‐in *post hoc* test for multiple comparisons to find significant differences between treatments. ***P < 0.005 vs. vehicle. # *P* < 0.05, ## *P* < 0.01, ### *P* < 0.005 vs. 5 μM Aβ_42_. Aβ = β‐amyloid; MaR1 = maresin 1

**Figure 3:**
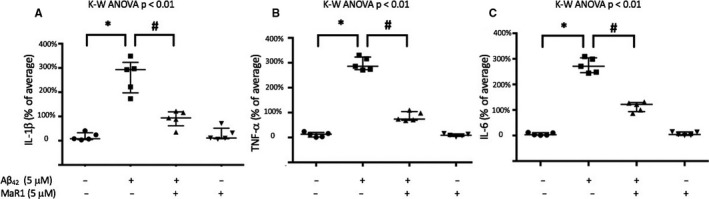
(A‐C) MaR1 reduced Aβ_42_‐induced pro‐inflammatory cytokine secretion in human MdM. Human monocyte‐derived microglia (MdM) were incubated with vehicle, 5 µM Aβ_42_, 5 µM MaR1 or 5 µM Aβ_42_ + 5 µM MaR1 for 24 h. The levels of interleukin (IL)‐1β (A), tumour necrosis factor (TNF)‐α (B) and IL‐6 (C) were determined in the cell supernatants using a V‐PLEX human pro‐inflammatory panel. A total of 5 experiments were performed. Aβ_42_ increased the levels of IL‐1β (A), TNF‐α (B) and IL‐6 (C), while co‐incubation with MaR1 reduced the elevation (A‐C). Analysis of variance (ANOVA) was performed with the Kruskal–Wallis (K‐W) test followed by pair‐wise comparisons of groups using Mann‐Withney with Bonferroni correction for multiple comparisons. *P < 0.05 vs. vehicle. # *P* < 0.05 vs. 5 μM Aβ_42_. Aβ = β‐amyloid; MaR1 = maresin 1

Aβ_42_ significantly increased the secretion of TNF‐α, IL‐1β and IL‐6 in both d‐THP‐1 cells (Figure 2A‐C) and MdM (Figure 3) (*P* values < 0.05). Co‐incubation with MaR1 significantly reduced the Aβ_42_‐induced secretion of TNF‐α, IL‐1β and IL‐6 (all *P* values < 0.01) (Figure 2 A‐C and Figure 3). MaR1 treatment alone did not alter the baseline expression of these cytokines (Figure 2A‐C and Figure 3). The anti‐inflammatory cytokines IL‐4 and IL‐10 were not detectable in d‐THP‐1 media, and neither was the soluble IL‐1 receptor type II. The levels of IL‐6Rα was neither affected by Aβ_42_ nor by MaR1 (Figure 2D).

### 3.3 MaR1 increased Aβ_42_‐uptake

**Figure 4:**
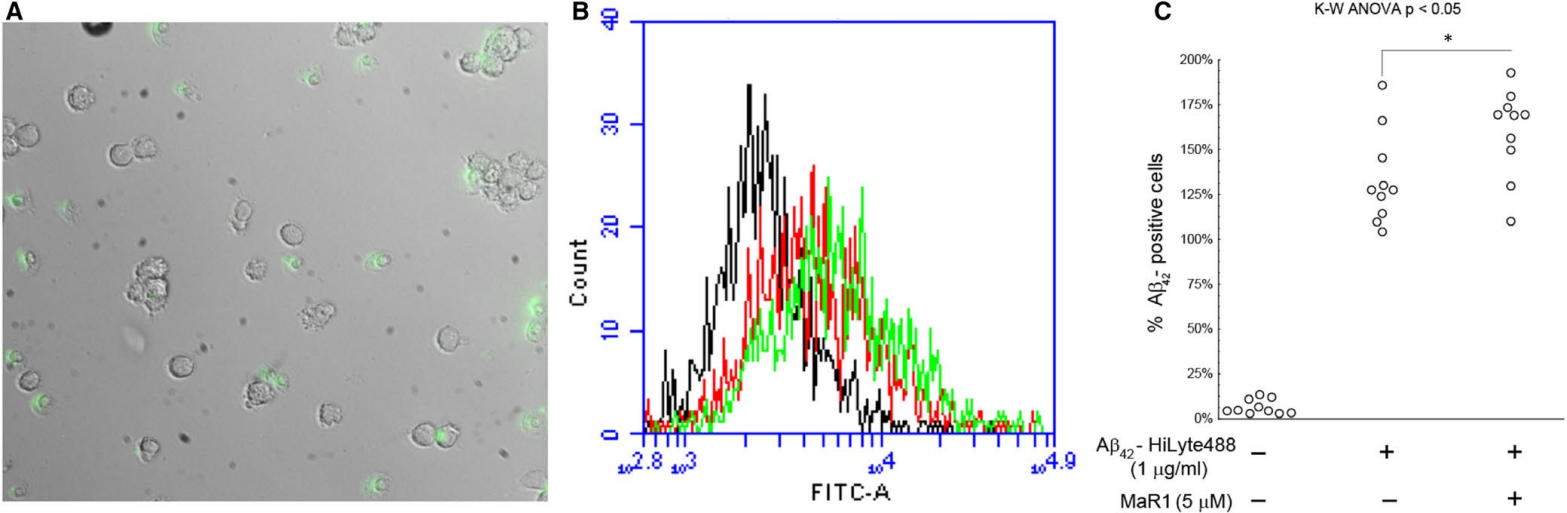
(A‐C) MaR1 increased Aβ_42_‐uptake in d‐THP‐1 cells. Differentiated THP‐1 (d‐THP‐1) cells were incubated for 20 min with 1 μg/mL HiLyteFluor 488‐conjugated Aβ_42_ alone together with 5 μM MaR1. Incubation with vehicle served as control. Aβ_42_ uptake was observed by fluorescence microscopy (A) and assessed by flow cytometry (B and C). Green fluorescence can be seen inside the cells indicating the uptake of Aβ_42_ (A). (B) shows an example of flow cytometry data from one experiment. For each treatment, one thousand gated events were analysed in the FITC‐channel. Vehicle control (black line) was considered as Aβ_42_‐negative. A right shift of the peak was observed upon incubation with Aβ_42_ indicating Aβ_42_‐uptake (red line), and MaR1 treatment further increased the Aβ_42_‐uptake (green line). Analysis of the data from ten experiments showed that MaR1 significantly increased the Aβ_42_‐uptake in d‐THP‐1 cells (C). Analysis of variance (ANOVA) was performed with the non‐parametric Kruskal–Wallis (K‐W) test, using the built‐in *post hoc* test for multiple comparisons to find significant differences between treatments. *P < 0.05 vs. 1 μg/mL Aβ_42_. Aβ = β‐amyloid; FITC = fluorescein isothiocyanate; MaR1 = maresin 1

To visualize the uptake of Aβ_42_ the d‐THP‐1 cells were incubated with 5 μg/mL HiLyteFluor 488‐conjugated Aβ_42_ for 20 minutes. A fluorescent signal could be seen within the cells (Figure 4A), indicating uptake of Aβ_42_. In order to investigate whether MaR1 could increase this uptake, the d‐THP‐1 cells were incubated for 20 minutes with 1 μg/mL HiLyteFluor 488‐conjugated Aβ_42_ alone or together with 5 µmol/L MaR1. The effect of MaR1 was analysed by flow cytometry of harvested cells from a total of 10 experiments. Uptake of Aβ_42_ was observed (Figure 4B) and the co‐incubation with MaR1 resulted in a statistically significant increase in this uptake (*P* < 0.05) (Figure 4C).

**Figure 5:**
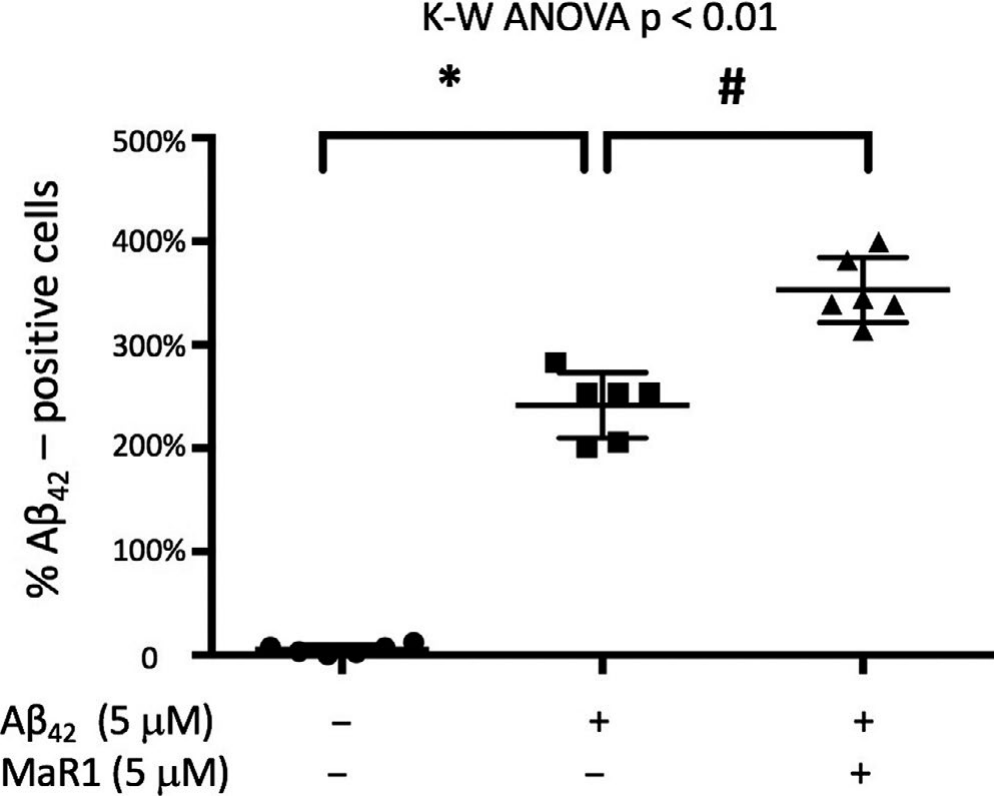
MaR1 increased Aβ_42_‐uptake in human MdM. Human monocyte‐derived microglia (MdM) were incubated with 1 μg/mL HiLyteFluor 488‐conjugated Aβ_42_ alone or together with 5 μM MaR1 for 90 min. Aβ_42_ uptake was assessed by flow cytometry. Co‐incubation with MaR1 significantly increased the uptake of Aβ_42_. Analysis of variance (ANOVA) was performed with the Kruskal–Wallis (K‐W) test followed by pair‐wise comparisons of groups using Mann‐Withney with Bonferroni correction for multiple comparisons. *P < 0.05 vs. vehicle. # *P* < 0.05 vs. 5 μM Aβ_42_. Aβ = β‐amyloid; MaR1 = maresin 1

Uptake of Aβ_42_ by MdM was analysed after incubation with 1 μg/mL HiLyteFluor 488‐conjugated Aβ_42_ for 90 minutes (Figure 5). The co‐incubation with MaR1 significantly increased the uptake (*P* < 0.05) (Figure 5).

### 3.4 MaR1 reduced Aβ_42_‐induced cell death

**Figure 6:**
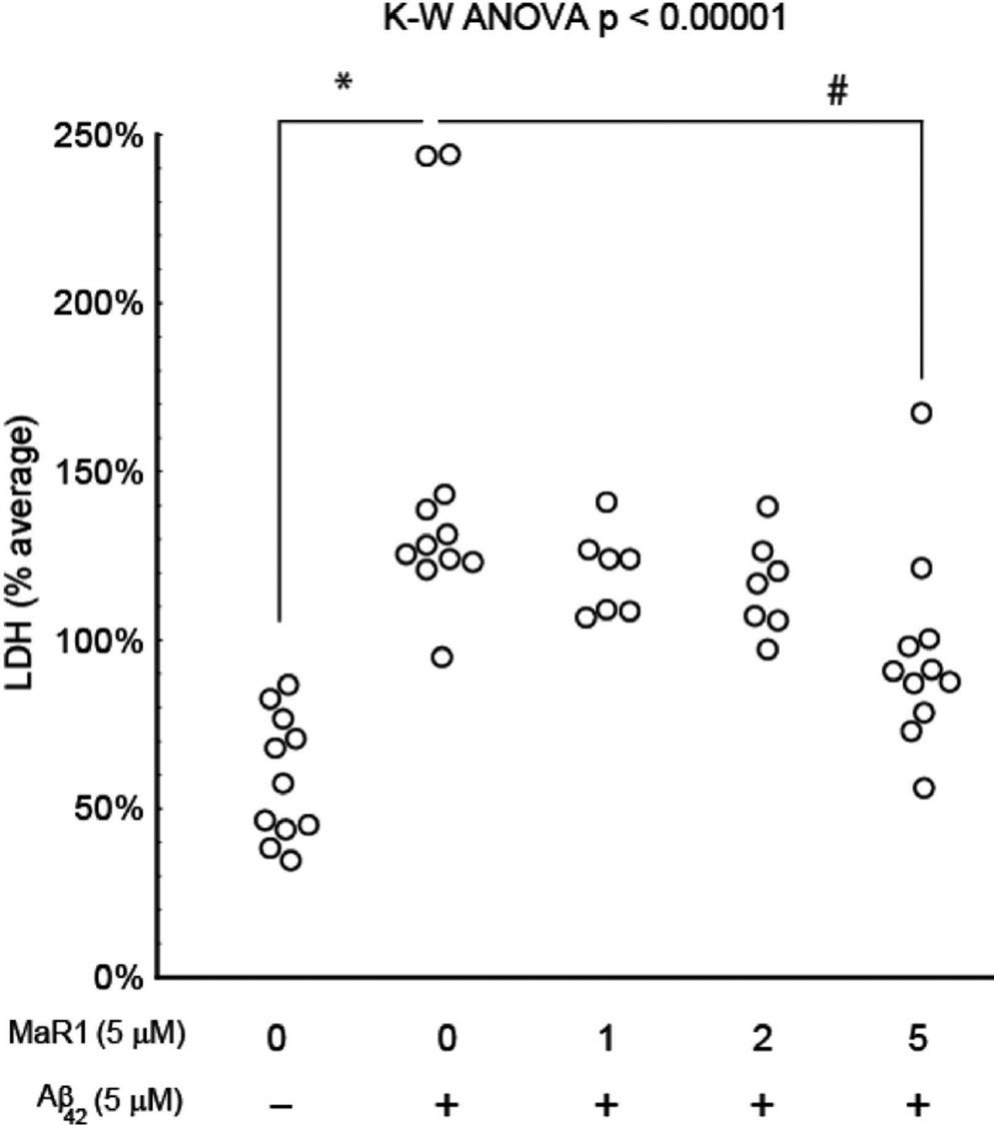
MaR1 reduced Aβ_42_‐induced cell death. Differentiated THP‐1 (d‐THP‐1) cells were incubated for 24 h with vehicle or with 5 μmol/L Aβ_42_ alone or together with 1, 2 or 5 μM MaR1. The cell culture supernatants were collected and 11 experiments were performed. Analysis by LDH assay showed an increase in cell death after incubation with Aβ_42_ (*P* < 0.0001). The co‐incubation with 5 μM MaR1 resulted in a reduction in Aβ_42_‐induced cell death (*P* < 0.05). Analysis of variance (ANOVA) was performed with a the non‐parametric Kruskal–Wallis (K‐W) test, using the built‐in *post hoc* test for multiple comparisons to find significant differences between treatments. *P < 0.05 vs. vehicle. ### *P* < 0005 vs. 5 μMAβ_42_. Aβ = β‐amyloid; LDH = lactate dehydrogenase; MaR1 = maresin 1

The effect of MaR1 on cell survival in d‐THP‐1 cells incubated with Aβ_42_ was analysed by the lactate dehydrogenase (LDH) assay. The cells were incubated for 24 hours with 5 μM Aβ_42_ alone or together with 0‐5 μM MaR1. Cell supernatants were collected in a total of 11 experiments. Aβ_42_ increased cell death 3‐fold, and MaR1 at a concentration of 5 μmol/L significantly reduced the cytotoxic effect of Aβ_42_ by approximately 30% (*P* < 0.05) (Figure 6).

### 3.5 MaR1 decreased pro‐inflammatory surface biomarkers

**Figure 7:**
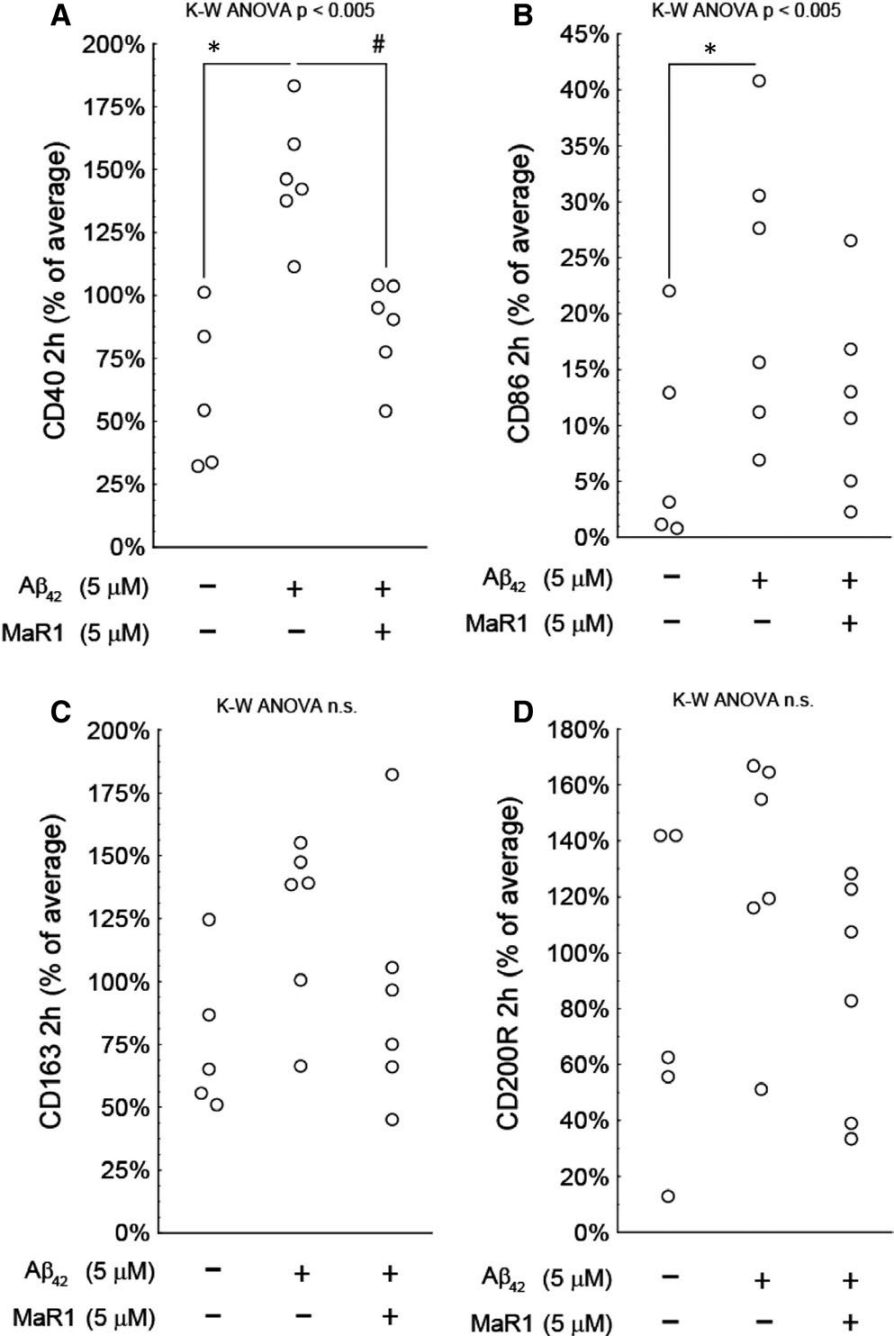
(A‐D) MaR1 reduced pro‐inflammatory surface biomarkers. Differentiated THP‐1 (d‐THP‐1) cells were incubated for 2 h with 5 μM Aβ_42_ alone or together with 5 μM MaR1. Incubation with vehicle served as control. The percentage of cells expressing pro‐ and anti‐inflammatory surface biomarkers CD40, CD86, CD163 and CD200R was assessed by flow cytometry in a total of six individual experiments. Incubation with Aβ_42_ significantly increased the pro‐inflammatory biomarkers CD40 and CD86. MaR1 attenuated the increase in CD40 (*P* < 0.05), whereas the anti‐inflammatory markers CD163 or CD200R were not affected by Aβ_42_ nor by Aβ_42_ + MaR1. Analysis of variance (ANOVA) was performed with the non‐parametric Kruskal–Wallis (K‐W) test, using the built‐in *post hoc* test for multiple comparisons to find significant differences between treatments. ***P < 0.005 vs. vehicle. # *P* < 0.05 vs. 5 μM Aβ_42_. Aβ = β‐amyloid, MaR1 = maresin 1

In order to investigate whether MaR1 could alter the phenotype of d‐THP‐1 cells in the context of AD, cells were incubated for 2 hours with 5 μM Aβ_42_ alone or together with 5 μM MaR1, followed by analysis of the pro‐inflammatory (CD40 and CD86) and anti‐inflammatory (CD163 and CD200R) biomarkers using flow cytometry (Figure 7). A total of six experiments were performed. Aβ_42_ significantly increased the expression of CD40 and CD80 about 3‐ and 2‐fold, respectively (Figure 7A and B), whereas there was no significant effect on CD163 nor CD200R (Figure 7C and D). MaR1 significantly reduced the Aβ_42_‐induced increase in CD40, nearly to baseline level (*P* < 0.05) (Figure 7A), whereas no effect of MaR1 was observed on the Aβ_42_‐induced levels of CD86 (Figure 7B).

### 3.6 MaR1 decreased Aβ_42_‐induced chemokine secretion

**Figure 8:**
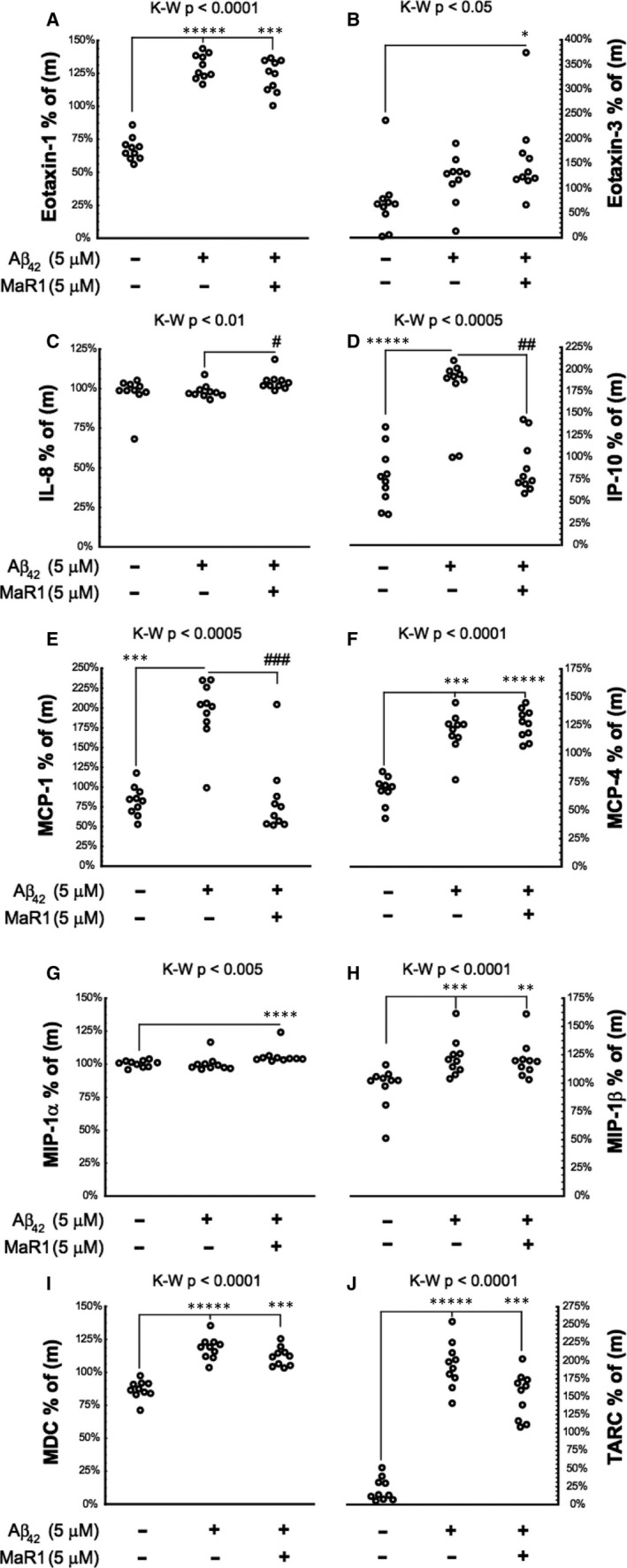
(A‐J) MaR1 reduced Aβ_42_‐induced chemokine secretion. Differentiated THP‐1 (d‐THP‐1) cells were incubated with 5 µM Aβ_42_ alone or together with 5 µM MaR1 for 24 h and analysed using a MesoScale 10‐plex human chemokine panel. Incubation with vehicle served as control. A total of ten experiments were performed. The chemokine data from each experiment were normalized to the average of the data from that experiment (m) and are presented in the Y‐axis in the graphs. MaR1 reduced the Aβ_42_‐induced increase in levels of interferon γ‐induced protein 10 (IP‐10/CXCL10) (D) and monocyte chemoattractant protein‐1 (MCP‐1/CCL2) (E), whereas the Aβ_42_‐induced increase in eotaxin‐1 (CCL11) (A), MCP‐4 (/CCL13) (F), macrophage inflammatory protein‐1β (MIP‐1β/CCL4) (H), macrophage‐derived chemokine (MDC/CCL22) (I) and thymus and activation regulated chemokine (TARC/CCL17) (J), was not affected by co‐incubation with MaR1. The levels of eotaxin‐3 (/CCL26) (B), interleukin (IL)‐8 (C) and MIP‐1α/CCL3 (G) were not increased by Aβ_42_. Analysis of variance (ANOVA) was performed with a non‐parametric Kruskal–Wallis (K‐W) test, using the built‐in *post hoc* test for multiple comparisons to find significant differences between treatments. *P < 0.05, **P < 0.01, ***P < 0.005, ****P < 0.001, *****P < 0.0005 vs. vehicle. #*P* < 0.05, ##*P* < 0.01, ### *P* < 0.005 vs. 5 μmol/L Aβ_42_. Aβ = β‐amyloid; MaR1 = maresin 1

The effect of MaR1 on the levels of Aβ_42_‐induced secretion of chemokines was investigated. The d‐THP‐1 cells were incubated for 24 hours with 5 μM Aβ_42_ alone or together with 5 μM MaR1 (Figure 8). At the end of the experiments (10 in total), the supernatants were collected for assessment of chemokines using a MesoScale 10‐plex human chemokine panel. Incubation with Aβ_42_ significantly increased eotaxin‐1 (Figure 8A), IP‐10 (Figure 8D), MCP‐1 (Figure 8E), MCP‐4 (Figure 8F), MIP‐1β (Figure 8H), MDC (Figure 8I), and TARC secretion (Figure 8J) (all *P* values < 0.001), while the levels of eotaxin‐3 (Figure 8B), IL‐8 (Figure 8C) and MIP‐1α (Figure 8G) were not affected. Co‐incubation with MaR1 significantly reduced the Aβ_42_‐induced increase in IP‐10 and MCP‐1 (Figure 8D and E) (all *P* values < 0.01), while no effect of MaR1 was seen on the other markers.

### 3.7 MaR1 decreased Aβ_42_‐induced NF‐κB activation

**Figure 9:**
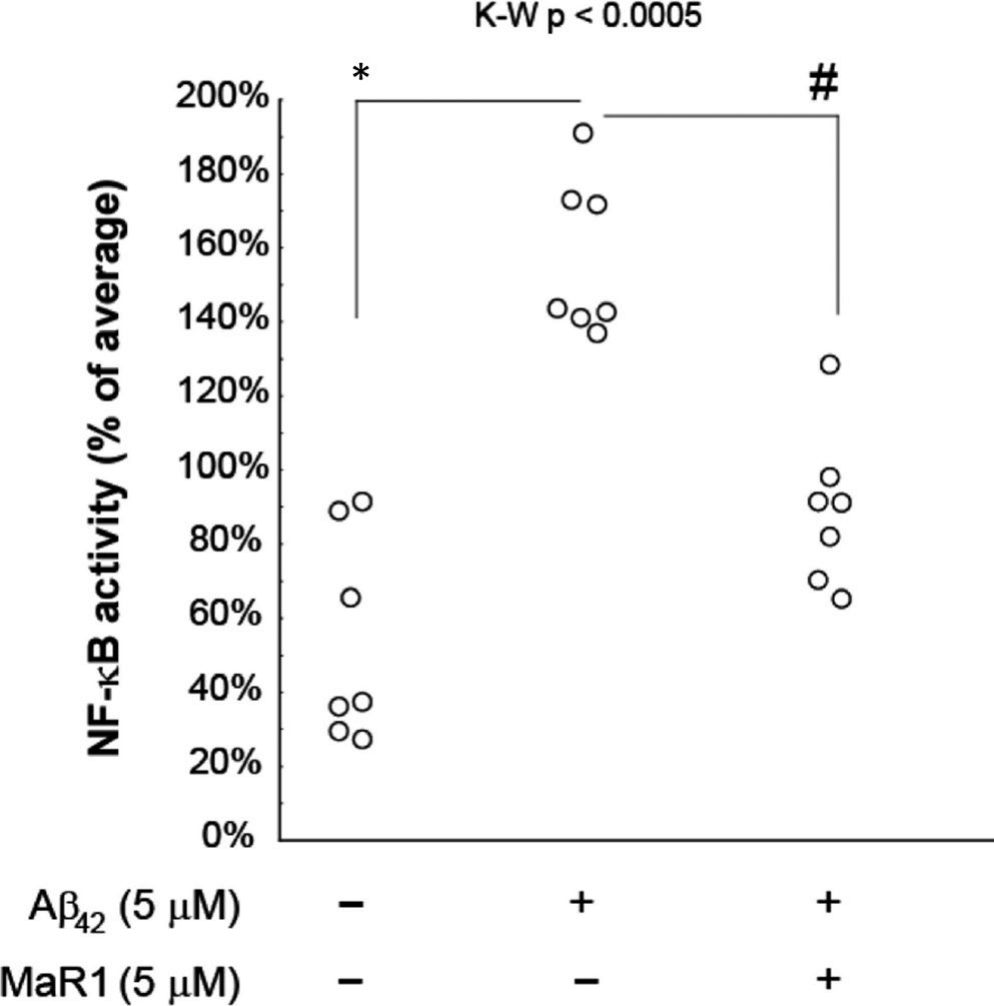
MaR1 reduced Aβ_42_‐induced NF‐κB activation. Differentiated THP‐1 (d‐THP‐1) cells were incubated with 5 µM Aβ_42_ alone or together with 5 µM MaR1 for 24 h, after which the luminescence in the conditioned medium was measured. Incubation with vehicle served as control. Co‐incubation with MaR1 showed a significant reduction of the Aβ_42_‐induced increase in NF‐κB activation. Analysis of variance (ANOVA) of the data was performed using the non‐parametric Kruskal–Wallis (K‐W) test. The built‐in *post hoc* test for multiple comparisons was used to find significant differences between treatments. *P < 0.05 vs. vehicle. #*P* < 0.05 vs. 5 μmol/L Aβ_42_. Aβ = β‐amyloid; MaR1 = maresin 1

To analyse the effect of MaR1 on NF‐κB activation, an NF‐κB reporter cell line based on THP‐1 cells was differentiated in the same manner as the non‐reporter cells and incubated with 5 µM Aβ_42_ alone or together with 5 µM MaR1 for 24 hours, after which the luminescence was analysed in the conditioned medium from a total of 7 experiments. Aβ_42_ increased the NF‐κB activity 3‐fold (*P* value < 0.01) and co‐incubation with MaR1 reduced this elevation by approximately 50% (*P* value < 0.05) (Figure 9).

### 3.8 MaR1 did not affect Aβ_42_‐induced kinase activation

The influence of MaR1 on the phosphorylation of key kinases of inflammatory pathways, including p38 MAPK, p44/42 MAPK, Akt and JNK was investigated by incubation of d‐THP‐1 cells for 24 hours with different concentrations of Aβ_42_ (0‐5 μM) alone or together with 5 or 10 μMMaR1. The phosphorylation of kinases was evaluated by Western blot and depicted by the ratio of the median signal intensity between phosphorylated and total kinase (See Figure S1). The analysis showed that the phosphorylation of p38 MAPK was increased by 2 or 5 μM Aβ_42_, whereas no effect of MaR1 was observed.

## 4 DISCUSSION

While many pro‐resolving functions have been ascribed MaR1, little is known about alterations in the levels of MaR1 in neurological diseases with an inflammatory component, and the implications for a decrease in the levels of MaR1 in these pathologies. Furthermore, the signalling mechanisms that transduce the pro‐resolving effects of MaR1 in the brain, and how they may be altered in neurodegenerative conditions remain unknown. These gaps in our knowledge need to be filled before considering clinical trials with this promising compound in humans. Although microglia‐mediated inflammation has been addressed in AD^5^, ^6^, ^7^ and decreased SPM levels have been observed in AD brains,^20^, ^22^, ^23^ there is a need to untangle the relationship between inflammation and SPMs in more detail. In the present study, using Aβ_42_‐treated MdM and d‐THP‐1 cells as in vitro models of microglia in AD, MaR1 was shown to have protective effects, including reduced secretion of pro‐inflammatory cytokines and chemokines, improving cell survival, and the attenuation of Aβ_42_‐induced NF‐κB activation.

To investigate neuroinflammation on a mechanistic level relevant and methodologically practical in vitro models are needed. As human primary microglia are difficult to obtain, other alternatives need to be considered. The two human in vitro microglial models used in this study have different *pros* and *cons*. THP‐1 cells, a human leukaemia monocytic cell line, are converted to macrophages by differentiation. Although microglia and macrophages are not the same, they share many properties, and microglia are often regarded as ‘brain macrophages’. For example, both microglia and macrophages surveil the tissue and maintain it by phagocytosis of debris and providing trophic support. Upon detection of a threat, macrophages as well as microglia become morphologically and biochemically activated and produce pro‐ and anti‐inflammatory cytokines, oxidative stress molecules, *etc* Many studies have indicated that THP‐1 cells and microglia respond to Aβ in a similar pattern, and therefore Aβ‐treated THP‐1 cells are frequently used to study AD‐like neuroinflammation.^7^, ^38^, ^39^, ^40^, ^41^ The parameters analysed in the present study represent functions expressed in macrophages as well as microglia, supporting the conclusions made. The major advantage of using a cell line such as THP‐1 is the unlimited supply of material. MdM is a more relevant model based on monocytes isolated from the blood which express a microglial phenotype after differentiation with a combination of cytokines, showing more similarity to human primary microglia than iPSC‐derived microglia, mouse primary microglia and human microglial cell lines,^42^ thus being the most relevant human in vitro model practically available. The disadvantage of using the MdM model is to obtain sufficient numbers of cells needed for assays such as Western blot, which is not a problem when using a cell line such as THP‐1.

Neuroinflammation is one of the key signatures of AD pathology. Cytokine levels are altered in CSF and plasma samples from patients with AD and mild cognitive impairment (MCI),^43^, ^44^ correlating to neuronal loss and memory deficits.^5^ We found that 5 μM MaR1 reduced Aβ_42_‐induced pro‐inflammatory cytokines. In two recent studies, the LPS‐induced release of TNF‐α and other pro‐inflammatory cytokines from monocytes (primary and cell line) was decreased by MaR1.^19^, ^45^ As MaR1 shows similar beneficial effects in various other models, such as spinal cord injury,^46^ obesity,^27^ liver injury ^29^ and acute lung injury,^47^ this indicates a general pro‐homeostatic effect in the organism.

Chemokines guide microglial migration to inflammatory areas and enhance the neuroinflammation in AD.^48^ The release of chemokines from microglia is up‐regulated upon Aβ stimulation.^49^ We found that 5 μM MaR1 reduced the Aβ_42_‐induced secretion of IP‐10 and MCP‐1. These data support the hypothesis that MaR1 reduces the Aβ_42_‐induced migration of inflammatory cells to areas with amyloid pathology. Recently, the treatment with 10 μM MaR1 was found to decrease the secretion of MCP‐1 from THP‐1 cells induced by LPS.^19^ The effect of MaR1 to normalize chemokine gradients has been observed in other models, including insulin sensitivity and MCP‐1 gene expression in obese mice,^28^ and LPS‐induced acute lung injury and levels of pro‐inflammatory cytokines and the chemokines MCP‐5, MIP‐1α and MIP‐1γ.^31^

Our studies show that MaR1 reduced pro‐inflammatory surface biomarkers. Thus, the Aβ_42_‐induced increase in CD40 was reduced almost to baseline levels by MaR1, supporting that the phenotype of microglia may have changed from pro‐inflammatory to pro‐resolving, in line with our earlier findings that MaR1 reduced Aβ_42_‐induced CD11b in human CHME‐3 microglia.^20^ Gone *et al* reported that MaR1 attenuated elevation of the pro‐inflammatory surface marker CD24 in the acute lung injury mice model induced by LPS.^31^

In order to investigate the down‐stream mediators of MaR1, we analysed the effects on NF‐κB activation, and found that it reduced Aβ‐induced NF‐κB activation, similarly to effects seen in other disease models.^26^, ^50^, ^51^, ^52^ This may be one of the mechanisms for the beneficial effects of MaR1 since NF‐κB is a transcription factor for many inflammatory genes and for amyloid precursor protein (APP).^53^ In order to further analyse the signal transduction mechanisms for the activities of MaR1, we analysed certain kinases related to pro‐inflammatory activation. However, p38 phosphorylation induced by Aβ_42_ was not affected by MaR1. This is in contrast to studies on other cellular models using activating stimuli other than Aβ_42_.^30^, ^46^ However, similarly to our findings, Gu et al found no evidence that the effects of MaR1 on LPS‐stimulated monocytes were mediated by a decrease in phosphorylation of the activating sites of p38 MAPK, p44/42 MAPK, JNK or Akt.^45^

We show that MaR1 increased Aβ_42_‐uptake, which is a proposed therapeutic strategy for AD that is associated with the past and present clinical trials based on treatment with anti‐Aβ antibodies. Using SPMs to stimulate Aβ‐uptake by microglia to achieve Aβ removal from the extracellular space and subsequent degradation may be a safer alternative than passive or active immunization, which in some cases has been linked to severe side effects.^54^ Supporting evidence for SPMs to reduce Aβ_42_ burden in vivo comes from studies on an AD mouse model in which co‐administration of two SPMs significantly reduced brain amyloid levels.^55^

Interestingly, MaR1 reduced Aβ_42_‐induced cell death of microglia, suggesting that MaR1 may exert a general cytoprotective function, that is not only on neurons, as described previously in a model of staurosporine‐induced neurotoxicity in differentiated human SH‐SY5Y neuroblastoma cells^20^ and by suppressing stress‐induced motor neuron cell death.^56^

The anti‐inflammatory cytokines IL‐4 and IL‐10 were not detected in the culture medium and other studies on THP‐1 cells have shown varying results.^57^, ^58^ While we have observed an increase in Aβ_42_‐uptake by MaR1 treatment, the degradation of Aβ_42_ will be an important focus for future studies aimed at investigating the potential of MaR1 as treatment for AD.

The present in vitro studies contribute to the understanding of how resolution of inflammation could be modulated in AD. SPMs have the advantage of being endogenously produced in humans, thereby reducing the risk of side effects. Protective effects of SPMs have been observed in neurological disease mouse models, including for AD,^55^ diffuse brain injury^59^ and in our recent study on a mouse model for Down Syndrome.^60^ These studies employing peripheral administration of SPMs indicate effective transfer across the blood–brain barrier.

In conclusion, our results strengthen the hypothesis that SPMs are protective and pro‐homeostatic compounds with the potential of having disease‐modifying, or even curative, effects in AD, and other neurodegenerative disorders that are characterized by a prominent inflammatory component. Hopefully, this new strategy for treating AD can lead to new treatments in the future, a prospect promoted by the fact that SPMs are endogenous compounds of no known toxicity.

## CONFLICT OF INTEREST

The authors declare that there are no conflicts of interest with regard to this study and its publication.

## Supporting information

Fig S1

Table S1

Table S2
