# Supplementary material for: Maresin 1 attenuates pro‐inflammatory activation induced by β‐amyloid and stimulates its uptake
Source: J Cell Mol Med. 2020 Nov 22;25(1):434–47. doi: 10.1111/jcmm.16098 (PMC7810927; doi:10.1111/jcmm.16098)
Supplement: Supplementary file 1 — Fig S1 [file JCMM-25-434-s001.docx]

**
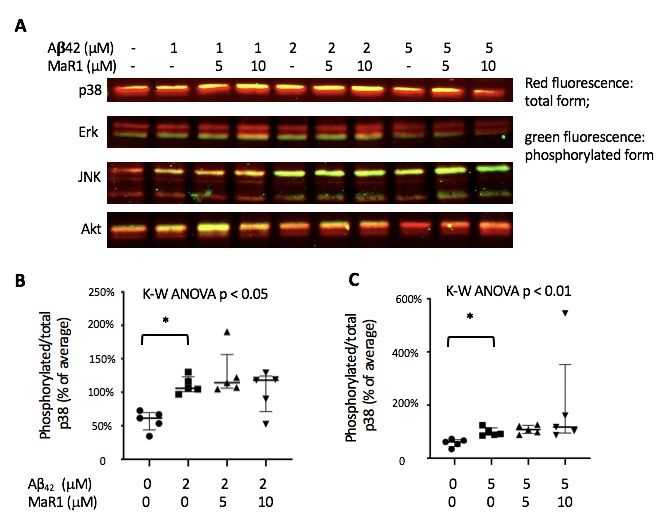
**

**Supplement Fig. 1. MaR1 did not affect Aβ_42_-induced kinase activation.** Differentiated THP-1 (d-THP-1) cells were incubated for 24 h with 1, 2 and 5 μM Aβ_42_ alone or together with 5 or 10 μM MaR1, and the phosphorylation of kinases was evaluated by Western blot (A). The data were obtained by calculating the ratio between the median signal intensity for each phosphorylated form and the corresponding intensity of total forms. The phosphorylation of p38 MAPK was increased by 2 (B) and 5 (C) μM Aβ_42_, whereas no effect of MaR1 was observed. Analysis of variance (ANOVA) was performed with the Kruskal-Wallis (K-W) test followed by comparisons between groups by Mann-Whitney U-test with Bonferroni correction for multiple comparisons. The data are presented by medians with quartile. * p < 0.05. Aβ = β-amyloid; p38 = p38 mitogen-activated protein kinase (MAPK); Erk = p44/42 MAPK; JNK = c-Jun N-terminal kinase; Akt = protein kinase B; MaR1 = maresin 1
