# Supplementary material for: Maresin 1 attenuates pro‐inflammatory activation induced by β‐amyloid and stimulates its uptake
Source: J Cell Mol Med. 2020 Nov 22;25(1):434–47. doi: 10.1111/jcmm.16098 (PMC7810927; doi:10.1111/jcmm.16098)
Supplement: Supplementary file 2 — Table S1 [file JCMM-25-434-s002.docx]

**Supplement Table 1. Test for lipopolysaccharide (LPS) contamination in purified Aβ**

| **Standards and samples** | **Optical density (OD)** | **LPS concentration (ng/ml)** |
| --- | --- | --- |
| Standard 1 | 0.128 | 200 |
| Standard 2 | 0.166 | 100 |
| Standard 3 | 0.247 | 50 |
| Standard 4 | 0.359 | 25 |
| Standard 5 | 0.520 | 12.5 |
| Standard 6 | 0.750 | 6.25 |
| Standard 7 | 0.959 | 3.13 |
| Blank | 1.295 | 0 |
| Met-Aβ from batch 1 | 1.132 | N/A |
| Met-Aβ from batch 2 | 1.127 | N/A |
| Met-Aβ from batch 3 | 1.085 | N/A |
| Met-Aβ from batch 4 | 1.184 | N/A |
| Wild-type Aβ from batch 1 | 1.153 | N/A |
| Wild-type Aβ from batch 2 | 1.135 | N/A |

Aβ = amyloid β; Met-Aβ = methionine-amyloid β
