# Supplementary material for: Maresin 1 attenuates pro‐inflammatory activation induced by β‐amyloid and stimulates its uptake
Source: J Cell Mol Med. 2020 Nov 22;25(1):434–47. doi: 10.1111/jcmm.16098 (PMC7810927; doi:10.1111/jcmm.16098)
Supplement: Supplementary file 3 — Table S2 [file JCMM-25-434-s003.docx]

**Supplement Table 2. Flow-cytometry antibodies**

| **Protein** | **Dilution** | **Isotypes** |
| --- | --- | --- |
| CD40 | 1: 25 | IgG1, κ |
| CD86 | 1: 50 | IgG2b, κ |
| CD163 | 1: 10 | IgG2b, κ |
| CD200R | 1:10 | IgG1, κ |

IgG = immunoglobulin
